# Rational Introduction of Electrostatic Interactions at Crystal Contacts to Enhance Protein Crystallization of an Ene Reductase

**DOI:** 10.3390/biom15040467

**Published:** 2025-03-22

**Authors:** Brigitte Walla, Anna Maslakova, Daniel Bischoff, Robert Janowski, Dierk Niessing, Dirk Weuster-Botz

**Affiliations:** 1 Biochemical Engineering, Department of Energy and Process Engineering, TUM School of Engineering and Design, Technical University of Munich, Boltzmannstraße 15, 85748 Garching, Germany; brigitte.walla@tum.de (B.W.); daniel.bischoff@tum.de (D.B.); 2 Molecular Targets and Therapeutics Center, Institute of Structural Biology, Helmholtz Zentrum München, Ingolstädter Landstraße 1, 85764 Neuherberg, Germanyniessing@helmholtz-muenchen.de (D.N.); 3 Institute of Pharmaceutical Biotechnology, Ulm University, James-Franck-Ring N27, 89081 Ulm, Germany

**Keywords:** downstream processing, protein isolation, crystal contact engineering, electrostatic interaction, stirred batch crystallizer, host cell protein (HCP)

## Abstract

Protein crystallization is an alternative to well-established but cost-intensive and time-consuming chromatography in biotechnological processes, with protein crystallization defined as an essential unit operation for isolating proteins, e.g., active pharmaceutical ingredients. Crystalline therapeutic proteins attract interest in formulation and delivery processes of biopharmaceuticals due to the high purity, concentration, and stability of the crystalline state. Although improving protein crystallization is mainly achieved by high-throughput screening of crystallization conditions, recent studies have established a rational protein engineering approach to enhance crystallization for two homologous alcohol dehydrogenases from *Lactobacillus brevis* (*Lb*ADH) and *Lactobacillus kefiri* (*Lk*ADH). As generalizing crystallization processes across a wide range of target proteins remains challenging, this study takes a further step by applying the successful crystal contact engineering strategies for *Lb*ADH/*Lk*ADH to a non-homologous protein, an NADH-binding derivative of the *Nostoc* sp. PCC 1720 ene reductase (*Nsp*ER1-L1,5). Here, the focus lies on introducing electrostatic interactions at crystal contacts, specifically between lysine and glutamic acid. Out of the nine tested *Nsp*ER1-L1,5 mutants produced in *E. coli*, six crystallized, while four mutants revealed an increased propensity to crystallize in static µL-batch crystallization compared to the wild type: Q204K, Q350K, D352K, and T354K. The best-performing mutant Q204K was selected for upscaling, crystallizing faster than the wild type in a stirred batch crystallizer. Even when spiked with *E. coli* cell lysate, the mutant maintained increased crystallizability compared to the wild type. The results of this study highlight the potential of crystal contact engineering as a reliable tool for improving protein crystallization as an alternative to chromatography, paving the way for more efficient biotechnological downstream processing.

## 1. Introduction

A comment on protein crystallization published in 2024 [1] announced a changing paradigm in industrial pharmaceutical crystallization, highlighting the importance of crystallization as a capture step in downstream processing with great economic potential (also reviewed in [2,3,4,5]). Advances in the understanding and control of crystallization mechanisms, as well as monitoring methods, help to transform crystallization into a rational and mechanism-based workflow for purification with the ability to control and monitor the incorporation of impurities in the final product [1,6,7,8,9,10]. The high purity and stability of crystalline biomolecules [11] have been attracting interest in therapeutic protein formulation and drug delivery/release for biomedical applications [12]. Biocompatible protein crystals also combine high drug concentration with low viscosity, which is advantageous for subcutaneous administration [13], e.g., by the reduced dosing frequency of crystalline insulin due to sustained release effects [14].

As protein crystallizability, crystal shape, and size depend on the proteins’ molecular properties in the crystallizing system, it is crucial to understand how these properties become apparent from molecular structures and intermolecular interactions [1]. Enormous progress has been achieved recently in the prediction of accurate models of biological complexes. For instance, AlphaFold2 has been explicitly trained for protein interaction prediction [15], and its successor AlphaFold3 offers even more advanced capabilities of high-accuracy prediction of complexes containing nearly all molecular types present in the Protein Data Bank (PDB) [16]. Nevertheless, it is still insufficiently understood how surface-exposed amino acids impact crystallizability, and this has thus been explored further lately [17,18,19,20]. Here, the alcohol dehydrogenase from *Lactobacillus brevis* (*Lb*ADH) was utilized as an example of a protein for technical protein crystallization studies to understand the impact of rational engineering strategies on crystallizability by single and double mutations, specifically at contact sites of the crystal lattice. Besides exchanging long flexible side chains (Lys, Arg) for shorter ones (Ala, Val), which is known as the surface entropy reduction (SER) strategy, developed by the group of Derewenda [21,22,23], aromatic interactions were presented to enhance crystallizability by introducing Tyr and Phe at a symmetrical crystal contact (D54F/Y) [19]. Enhancing electrostatic interactions by introducing charged amino acid residues (Glu, Lys, His) increased the crystallizability of *Lb*ADH for mutants Q126H [18] and T102E [19]. Increased/enhanced crystallizability was thereby defined (as described by Grob et al. [19]) for static and dynamic crystallization by (i) an extended nucleation window towards lower protein/crystallization agent concentrations, (ii) a higher amount of crystals at crystallization equilibrium, which is equivalent to a higher nucleation rate, (iii) a shorter induction time, (iv) a shorter period of crystal growth, and (v) a shorter time until crystallization equilibrium. These characteristics correlate with a higher space–time yield and lower usage of consumables (crystallization agent), which are relevant factors impacting the efficiency of technical crystallization as a purification step in downstream processing [24,25].

The introduction of electrostatic interactions as a strategy to engineer crystal contacts was successfully transferred to a homologous enzyme, the *Lactobacillus kefiri* alcohol dehydrogenases (*Lk*ADH, sequence homology: 88.5%), demonstrated in *Lk*ADH mutants T102E and Q126K [20]. As enzymatic activity is a substantial parameter in protein technologies, the *Lb*ADH/*Lk*ADH mutants published were positively tested for preserved function.

To generalize this engineering approach for crystal contacts, a non-ADH-homologous enzyme was selected to apply the established strategies to the *Nostoc* sp. PCC 1720 ene reductase (*Nostoc*ER). This oxidoreductase from the old yellow enzyme family (OYE, EC 1.6.99.1 [26]) catalyzes the *trans*-hydrogenations of activated alkenes [27,28], demonstrating its industrial relevance as a biocatalyst (reviewed in [27,29,30,31]). As OYEs are strongly dependent on cost-intensive NADPH as a cofactor [32], Mähler et al. engineered NADH-accepting variants (*Nostoc*ER1) by exchanging *Nostoc*ER loop regions at cofactor-binding sites to ER loop regions from *Achromobacter* sp. JA81 [33] and *Acaryochloris marina* [34] with a higher NADH affinity. The variants with the fastest and highest catalytic properties are *Nostoc*ER1 Loop1,2a, Loop1,5, and Loop1,5,2a [34]. For this study, the *Nostoc*ER1 Loop1,5 variant (*Nostoc*ER1-L1,5) was selected as an example protein for the rational introduction of Lys–Glu interactions at crystal contacts to demonstrate the impact of rational single amino acid exchanges on the crystallizability of proteins and enhance protein crystallization.

## 2. Materials and Methods

### 2.1. Site-Directed Mutagenesis

For applying successfully established crystal contact engineering strategies on alcohol dehydrogenases (ADH) from *Lactobacillus brevis* (PDB ID: 6H07) and *L. kefiri* (PDB ID: 7P36) to a non-homologous enzyme, *Nostoc* sp. PCC 7120 ene reductase (PDB ID: 6UFF [35]) was selected. The NADPH cofactor specificity of *Nostoc* sp. PCC 7120 ene reductase was modified by Mähler et al. [34] by changing two loop binding regions, resulting in an NADH-binding *Nostoc*ER1-L1,5. The N-terminal tag and linker sequence of *Nostoc*ER1-L1,5, encoded on pET28a(+), was shortened by 10 amino acids via Gibson assembly [36], aligning it to the ADH tags accordingly. The resulting sequence with an N-terminal His_6_ tag, followed by a glycine–serine–glycine (GSG) linker, was named *Nsp*ER1-L1,5 (PDB ID: 9QGB). To study the introduced Lys–Glu interactions specifically at crystal contacts, site-directed mutagenesis was performed to generate *Nsp*ER1-L1,5 mutants Q171E, Q204K, Q263K, A264K, D280K, V344E, Q350K, D352K, and T354K according to the standard QuikChange PCR protocol (developed by Stratagene, La Jolla, CA, USA). Partially overlapping primers were designed as adapted from Zheng et al. [37] and are listed in Supplementary Table S1. Plasmid amplification and mutant sequencing were performed according to Walla et al. [20].

### 2.2. Heterologous Protein Production and Processing

For heterologous protein production, the chemically competent *E. coli* strain BL21(DE3) was transformed with an *Nsp*ER1-L1,5 variant plasmid. Analogous to Nowotny et al. [18], 5 mL of terrific broth (TB) medium containing 35 µg mL^−1^ kanamycin were inoculated with a single colony and incubated overnight (min. 16 h, 180 rpm, 30 °C). For the main culture, 1 L shake flasks filled with 200 mL TB medium (35 µg mL^−1^ kanamycin) were inoculated with a preculture (starting OD_600_ of 0.05, 30 °C, 230 rpm). When an OD_600_ of 0.6–0.8 was reached, *Nsp*er gene expression was induced by adding 200 µM isopropyl β-D-1-thiogalactopyranoside (IPTG). After 20 h of *Nsp*ER1-L1,5 protein production at a reduced temperature (20 °C, 230 rpm), the cells were harvested by centrifugation (1500× *g*, 4 °C, 10 min) and stored at −20 °C for further use according to Walla et al. [20]. The thawed cell pellets on ice were resuspended in 10 mL 1× phosphate-buffered saline (PBS, pH 7.4) and disrupted via sonication (50% cycle, 90% intensity, 3 × 3 min); the *E. coli* cell lysate was centrifuged for clarification of the cell debris (12,000× *g*, 4 °C, 1 h). For crystallization and enzymatic activity measurements, the *Nsp*ER1-L1,5 protein was purified via a 2-step immobilized metal ion affinity chromatography (IMAC) according to Nowotny et al. [18]. The clarified *E. coli* cell lysate was loaded onto an equilibrated HisTrap HP column (1 or 5 mL; Cytiva, Chicago, IL, USA; ÄKTA Pure system, GE Healthcare Life Science, Munich, Germany), and the *Nsp*ER1-L1,5 protein was selectively eluted via its N-terminal His_6_ tag with 500 mM imidazole, 300 mM NaCl, and 10 mM Na_2_HPO_4_/NaH_2_PO_4_ (pH 7.5). The collected eluates were dialyzed against a protein buffer (150 mM NaCl, 25 mM Na_2_HPO_4_/NaH_2_PO_4_, pH 7.2) and stored at 4 °C until further use.

### 2.3. Static and Dynamic Protein Crystallization

Screening for crystallization conditions of the *Nsp*ER1-L1,5 (IMAC-purified and dialyzed against a protein buffer) was performed with two commercial crystallization screens (Index HT and Natrix HT, Hampton Research, Aliso Viejo, CA, USA) and evaluated after five days of incubation at 20 °C.

Parallel static µL-batch crystallization experiments were conducted with IMAC-purified and dialyzed protein solutions in 96-well micro-batch plates (MCR Under Oil Crystallization Plate, Jena Bioscience GmbH, Jena, Germany) according to Nowotny et al. [18]. The samples’ concentration was calculated spectrophotometrically at 280 nm with a theoretical molar extinction coefficient of 38,850 M^−1^ cm^−1^ (calculated with ProtParam, https://web.expasy.org/protparam/ [38]) and adjusted to similar concentrations with a protein buffer.

*Nsp*ER1-L1,5 mutant Q204K (3.75–10 g L^−1^) was also tested with two further crystallization conditions: 50 mM HEPES, 0.1 M NaCl, 150 g L^−1^ PEG 6000, pH 7.5 (HEPES crystallization buffer) and 50 mM Tris, 0.1 M ammonium acetate, 150 g L^−1^ PEG 6000, pH 8.5 (Tris crystallization buffer).

For dynamic protein crystallization, a stirred 5 mL crystallizer with pitched-blade impellers was used, as described by Smejkal et al. [39] and elaborated by Grob et al. [19] and Walla et al. [20]. Protein crystallization was initiated by adding 2.5 mL crystallization buffer (50 mM Tris/HCl, 0.2 M NH_4_Cl, 5 mM CaCl_2_, 250–400 g L^−1^ PEG 6000, pH 8.5) to the prepared protein solution, resulting in a protein concentration defined as the initial protein concentration c_0_, and monitored for 48 h with regular sampling intervals.

To examine the impact of the host cell protein (HCP) on crystallization, static crystallization was performed as described above, with a purified *Nsp*ER1-L1,5 solution (10 g L^−1^) combined with increasing amounts of HCP (0–70% (*w*/*v*)). For these static crystallization experiments and stirred crystallization in 5 mL reactors with 20% (*w*/*v*) HCP, the flow-through (non-binding, unspecific proteins of the *E. coli* cell lysate) was collected during IMAC and dialyzed accordingly (10 g L^−1^).

### 2.4. Protein Analytics

The protein purity of the collected IMAC fractions was validated by discontinuous sodium dodecyl sulfate–polyacrylamide gel electrophoresis (SDS-PAGE). The enzymatic activity of purified *Nsp*ER1-L1,5 variants (60 mg L^−1^) was determined spectrophotometrically as described by Mähler et al. [34] with adapted assay buffer component concentrations (0.2 mM NADH, 10 mM maleimide, 120 mM NaH_2_PO_4_/Na_2_HPO_4_, pH 7.0) at 30 °C. The oxidation of maleimide catalyzed by *Nsp*ER1-L1,5 was detected at 340 nm by stoichiometric reduction of NADH to NAD^+^ (measurements in quintuplicates within 10 min, every 6 s).

Analogous to Walla et al. [20], *Nsp*ER1-L1,5 protein concentration during stirred crystallization in 5 mL reactors was analyzed with bicinchoninic acid (BCA) protein assay and spectrophotometric measurements. A discontinuous SDS–PAGE (12.5% bisacrylamide in running gel, 300 V, 35 mA, 1 h [40,41]) was used to validate similar *Nsp*ER1-L1,5 protein concentrations between the samples. For crystal photomicrographs, samples were taken after 20.5 h of protein crystallization and diluted 2-fold. A 10 µL drop was placed in a crystallization plate and monitored microscopically inside an incubator (KB115, Binder, Tuttlingen, Germany) at 20 °C. Photomicrographs were taken automatically at defined time intervals, as described by Walla et al. [20].

### 2.5. X-Ray Diffraction, Data Refinement, and Structure Analysis

The crystal structure of the previously published *Nostoc*ER wild type (PDB ID: 6UFF) was used as a search model for molecular replacement with PHASER (v.2.8.3) [42]. The model was then refined using COOT (v.0.9.8) [43] and REFMAC (v.5.8) [44]. The final model and structure factors have been deposited in the Protein Data Bank (PDB) under identification code (ID) 9QGB for the *Nsp*ER1-L1,5 wild type and under the following IDs for the *Nsp*ER1-L1,5 mutants: Q204K (PDB ID: 9QGC), Q350K (PDB ID: 9QGD), D352K (PDB ID: 9QGE), and T354K (PDB ID: 9QGF). Quality indicators for the X-ray diffraction datasets and refinement results are given in Supplementary Table S2. The electron density maps in Supplementary Figure S2 were calculated with REFMAC [44] and represent the structure factor amplitude difference 2F_o_–F_c_ with a contour level of 1.0 σ.

The root-mean-square deviation (RMSD) of Cα atoms (Supplementary Table S3) compares the crystal packing of the protein chains contained in the asymmetric unit of the five *Nsp*ER1-L1,5 variants (protein chains are labeled by their chain identifiers). The RMSD values are calculated by finding the transformations that superpose each chain of each variant (columns) onto either chain A or chain B of the wild type (rows) in the asymmetric units using PyMOL (v.2.3) [45]. The crystalline environment of each chain and each variant is then constructed using all the protein chains within 10 Å using PyMOL. This crystalline environment is subsequently superposed on each of the wild type chains using the corresponding transformation obtained in the previous step. Finally, the RMSD of these oligomeric structures is calculated with US-align [46] by finding a chain ordering that minimizes the RMSD without an additional superposition step. This leads to very well-aligned central chains of the crystalline environments, with large RMSD values indicating structural differences in the crystal packing.

## 3. Results

### 3.1. Semirational Selection of Mutants by Evaluation of the NspER1-L1,5 Wild Type’s X-Ray Structure

To select positions at a crystal contact for potential engineering to enhance the protein’s crystallizability, a crystal structure with known crystallization conditions is needed. Furthermore, for this study, the reproducibility of protein crystallization in batch crystallization plates within a short period (<2 days) needed to be ensured. For this purpose, a high-throughput screening of crystallization conditions was performed with commercial screening plates, as described in Section 2.3. Out of the crystallization conditions resulting in crystals within two days, the following were selected based on cost efficiency and compatibility for customers and the environment: 25 mM Tris–HCl, 0.1 M NH_4_Cl, 5 mM CaCl_2_·2H_2_O, 50–150 g L^−1^ PEG 6000, pH 8.5. X-ray diffraction analysis performed on crystals taken from µL-batch experiments revealed a crystal structure with a resolution of 1.27 Å (see Section 2.5). Based on the observed intermolecular crystal contacts, nine amino acids were selected for *Nsp*ER1-L1,5 mutation (Table 1). They were selected based on their distance to a potential intermolecular interaction partner at the crystal contact of <6 Å (except for Q263K). This range was defined by the commonly known distance between residues participating in salt bridges of usually ≤4 Å, with an additional length due to the potential inaccuracy of in silico mutated model structures (generated using PyMOL (v.2.3 [45])).

**Figure 1:**
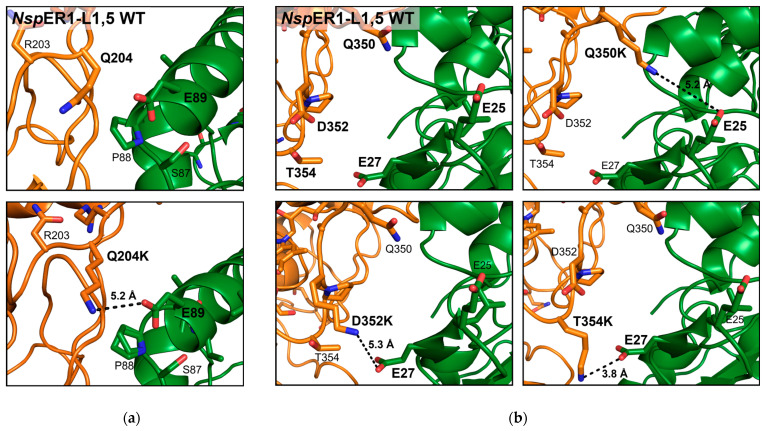
Illustration of the *Nsp*ER1-L1,5 WT crystal contact (PDB ID: 9QGB) at position 204 (**a**) and positions 350–352 (**b**) in the wild type and for the selected mutants. Mutants Q204K, Q350K, D352K, and T354K were generated *in silico,* and the distances to the potential interaction partners E89 and E25/27, respectively, were calculated using PyMOL (v.2.3 [45]).

Exemplarily, the *Nsp*ER1-L1,5 crystal contact at positions 204 and 350–353 is depicted below (Figure 1), with mutations Q204K and Q350K/D352K/T354K generated *in silico* and the distance between the potential interacting amino acid residues E89 and E25/27, respectively, calculated using PyMOL (v.2.3 [45]).

### 3.2. Static µL-Batch Crystallization of the Purified NspER1-L1,5 Variants

The selected *Nsp*ER1-L1,5 mutants listed in Section 3.1 (Table 1) were cloned, expressed, and processed as described in Section 2.1 and Section 2.2. The enzymatic activity of the *Nsp*ER1-L1,5 variants was verified spectrophotometrically (Section 2.4) and is shown in Supplementary Figure S1. The IMAC-purified and dialyzed *Nsp*ER1-L1,5 variant protein solutions were crystallized in µL-batch plates, as described in Section 2.3, with 50 mM Tris/HCl, 0.2 M NH_4_Cl, 5 mM CaCl_2_, 100–300 g L^−1^ PEG 6000, pH 8.5 as the crystallization buffer. Table 2 below lists the results of the µL-batch crystallization experiments with the *Nsp*ER1-L1,5 variants regarding crystallizability parameters as defined in the Introduction (crystallization start, duration until crystallization equilibrium, lowest crystallization conditions at which crystallization occurs). Photomicrographs of *Nsp*ER1-L1,5 mutants from six individual experiments were evaluated, with each experiment including the crystallization of the WT under the same conditions as the mutants for comparison.

Table 2 reveals that four out of the nine tested *Nsp*ER1-L1,5 mutants had a higher crystallizability compared to the WT in the µL batch regarding crystallization start and duration until crystallization equilibrium: Q204K, Q350K, D352K, and T354K. Mutant Q204K performed significantly better than the WT in two out of the three tested parameters, while mutants Q350K, D352K, and T354K excelled in one parameter (crystallization start was 2 h/3 h earlier, extended nucleation window, respectively). Mutants A264K and V344E did not crystallize under the tested conditions within 50 h.

**Figure 2:**
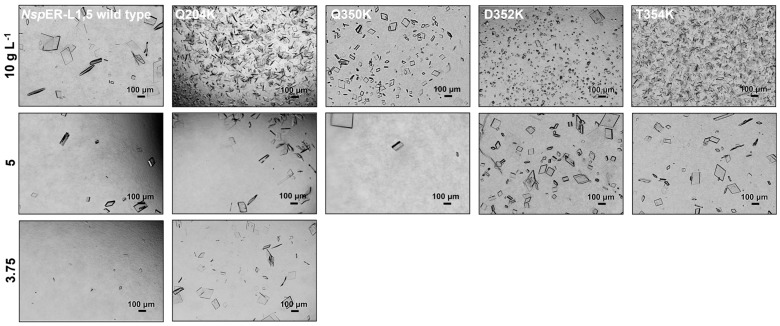
Crystallization photomicrographs of the *Nsp*ER1-L1,5 WT and mutants Q204K, Q350K, D352K, and T354K with crystallization conditions of 10 g L^−1^ (**top row**), 5 g L^−1^ (**middle row**), and *3.7*5 g L^−1^ (**bottom row**) purified protein, completed with a crystallization buffer (150 g L^−1^ PEG 6000). *Nsp*ER1-L1,5 mutants Q350K, D352K, and T354K were not tested with *3.7*5 g L^−1^. The crystallization mix was incubated at 20 °C, and photomicrographs were taken after 48 h.

For further validation of the mutants’ crystallizability regarding crystal size and amount, photomicrographs of the *Nsp*ER1-L1,5 WT and mutants Q204K, Q350K, D352K, and T354K taken after 45 h of crystallization are presented below (Figure 2).

The *Nsp*ER1-L1,5 WT and mutants Q204K, Q350K, D352K, and T354K crystallized under the crystallization condition containing 10 g L^−1^ of purified protein and 150 g L^−1^ PEG 6000 in the crystallization buffer, whereby the crystal amount was highest, and the crystal size was smallest for mutant T354K, followed by Q204K and D352K (Figure 2). Mutant Q350K crystallized similarly to the WT regarding crystal size and amount. Reducing the protein concentration from 10 g L^−1^ to 5 g L^−1^ led to a decrease in the crystal amount for all the tested *Nsp*ER1-L1,5 variants, with D352K being the mutant that formed crystals of the smallest size but in the highest amount. Additionally, reduced crystallization conditions with 3.75 g L^−1^ purified protein (150 g L^−1^ PEG 6000) were tested for Q204K and WT. Here, mutant Q204K crystallized similarly to the results with 5 g L^−1^, whereas only three small (<10 µm) WT crystals grew. The evaluation of the *Nsp*ER1-L1,5 variant photomicrographs indicated increased crystallizability for mutants Q204K, D352K, and T354K regarding the tested parameters of crystal size and amount. In addition, crystallization of 3.75 g L^−1^ Q204K revealed an extended nucleation window toward lower protein concentrations for this mutant.

**Figure 3:**
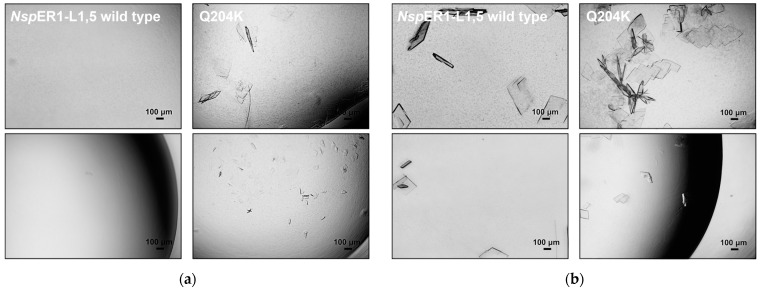
Crystallization photomicrographs of the *Nsp*ER1-L1,5 WT and mutant Q204K tested under extended crystallization conditions: (**a**) 50 mM HEPES, 0.1 M NaCl, pH 7.5 (HEPES crystallization buffer) and (**b**) 50 mM Tris, 0.1 M ammonium acetate, pH 8.5 (Tris crystallization buffer), completed with 150 g L^−1^ PEG 6000 and combined with 5 g L^−1^ (**top row**) and 10 g L^−1^ (**bottom row**) protein solution (1:1). The crystallization mix was incubated at 20 °C, and photomicrographs were taken after 48 h.

As *Nsp*ER1-L1,5 mutant Q204K stood out as the mutant with the highest crystallizability, two different buffers (HEPES and Tris crystallization buffers) were tested to further characterize the altered crystallizability, as depicted below (Figure 3).

The photomicrographs of the *Nsp*ER1-L1,5 WT and mutant Q204K crystallized in two different buffers (Figure 3) revealed the enhanced crystallizability of Q204K compared to the WT protein. Mutant Q204K crystallized in both the HEPES (Figure 3a) and Tris (Figure 3b) buffers at both protein concentrations tested (5/10 g L^−1^). However, for the *Nsp*ER1-L1,5 WT, no crystals were visible with the Tris buffer, and a lower number of larger crystals (compared to mutant Q204K) grew with the HEPES buffer.

### 3.3. NspER1-L1,5 Mutant Q204K: Scaling to Dynamic Crystallization in a 5 mL Crystallizer

**Figure 4:**
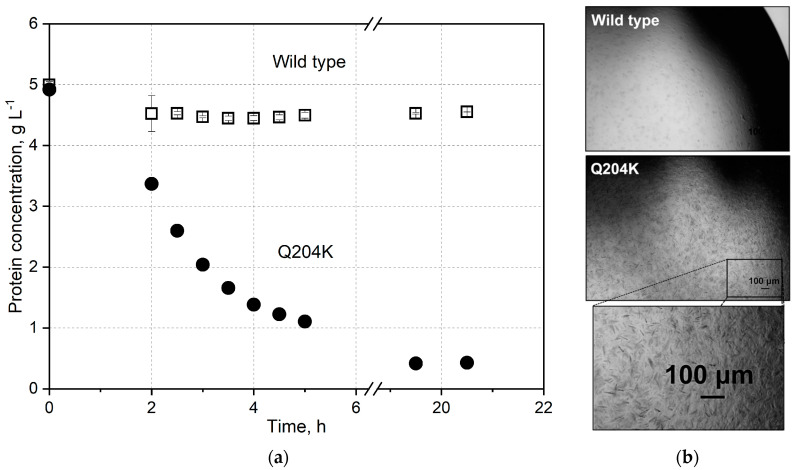
Stirred mL-batch crystallization of the purified *Nsp*ER1-L1,5 wild type and mutant Q204K with PEG 6000 as the crystallization agent. (**a**) Supernatant protein concentration of the *Nsp*ER1-L1,5 wild type and mutant Q204K during crystallization from an IMAC-purified protein solution in parallel stirred tank reactors (c_0_ = 5 g L^−1^, V = 5 mL, n_Sstirrer_ = 150 min^−1^, T = 20 °C, 250 g L^−1^ PEG 6000). (**b**) Photomicrographs of the *Nsp*ER1-L1,5 wild type and mutant Q204K taken after 20.5 h of crystallization (2-fold diluted with a protein buffer).

Dynamic batch crystallization studies in stirred 5 mL crystallizers were conducted to study protein crystallization as a downstream processing step for purification in technical applications. The dynamic *Nsp*ER1-L1,5 crystallization in a 5 mL crystallizer was established using an IMAC-purified and dialyzed (against the protein buffer) protein solution and a crystallization buffer (Section 2.3) with 200 g L^−1^ PEG 6000. *Nsp*ER1-L1,5 mutant Q204K was selected for the scaling studies in a 5 mL stirred crystallizer compared to the WT since Q204K stood out as the mutant with the highest crystallizability in µL-batch crystallization studies. The protein concentrations of the *Nsp*ER1-L1,5 WT and Q204K measured during the experiment are shown as a function of time below in Figure 4.

After crystallization initiation (addition of the crystallization buffer to the protein solution), the protein concentration of mutant Q204K declined and dropped to 1.1 g L^−1^ within five hours. Subsequently, the protein concentration of mutant Q204K decreased significantly slower, and the equilibrium was reached at 0.4 g L^−1^ within 19.5 h with a crystal yield of 91.7%. Contrary to the Q204K mutant, the addition of PEG 6000 did not initiate the *Nsp*ER1-L1,5 WT crystallization in a stirred 5 mL crystallizer. Its concentration in the liquid phase remained nearly constant throughout the process. The slight decrease in WT concentration from 5 g L^−1^ to 4.5 g L^−1^ observed after the addition of PEG 6000 could be caused by a dilution effect or submicroscopic aggregation.

In accordance with the protein concentration measurements (Figure 4a), no crystals were observed in the crystallization samples of the *Nsp*ER1-L1,5 WT after 20.5 h of crystallization (Figure 4b). However, the samples of mutant Q204K contained a high number of needle-like crystals after 20.5 h (Figure 4b), already starting to form 2 h after crystallization initiation.

**Figure 5:**
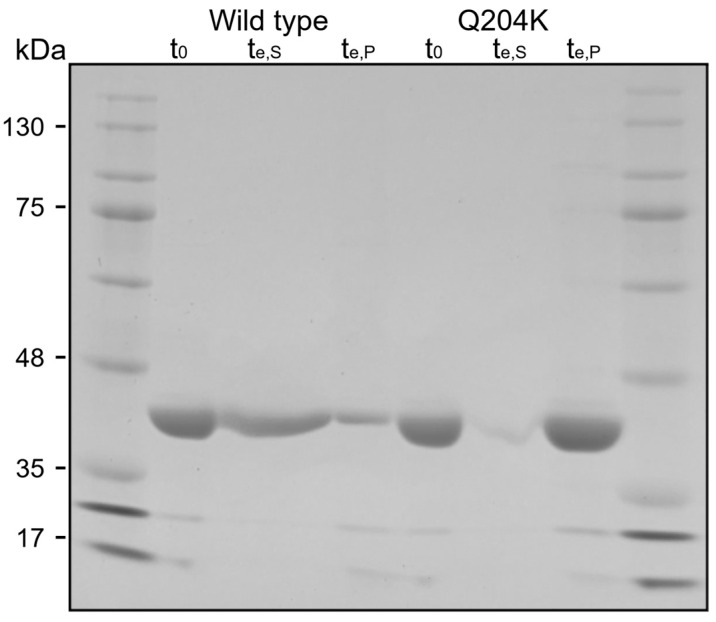
SDS–PAGE visualizing the *Nsp*ER1-L1,5 WT and mutant Q204K samples of stirred 5 mL-batch crystallization (300 V, 35 mA, 50 min). Depicted are samples directly before crystallization initiation (t_0_ = 0 h) and after 20.5 h (t_e_) of crystallization in stirred crystallizers (V = 5 mL, n_Stirrer_ = 150 min^−1^, 20 °C, 250 g L^−1^ PEG 6000). For t_e_, samples from the supernatant (t_e,S_) and the pellet (t_e,P_) were differentiated. *Nsp*ER1-L1,5 variants were detected between 35–48 kDa, corresponding to its molecular monomer weight of 42.98 kDa (calculated with ExPASy ProtParam). Marker: BlueStar Prestained Protein Marker (Nippon Genetics Europe GmbH, Dueren, Germany). SDS-PAGE can be found in Supplementary Materials.

The first (t_0_) and the last (t_e_) crystallization samples of the *Nsp*ER1-L1,5 WT and mutant Q204K, taken at 0 h and 21.5 h, respectively, were analyzed via SDS–PAGE, as depicted in Figure 5.

As shown in Figure 5, SDS–PAGE analysis confirmed the presence of a protein band with a molecular weight between 35 kDa and 48 kDa in the initial samples of the *Nsp*ER1-L1,5 WT and mutant Q204K (t_0_), corresponding to the molecular monomer weight of 42.98 kDa (calculated with ExPASy ProtParam). After 20.5 h, protein bands of the same molecular weight could be observed for the WT in the supernatant (t_e,S,WT_) and the pellet (t_e,P,WT_), as well as in the pellet sample of Q204K (t_e,P_). For the WT, the bandwidth of the supernatant sample (t_e,S,WT_) was significantly larger than that of the pellet sample (t_e,P,WT_), indicating a high amount of the WT protein remaining in the solution after the end of the crystallization process. On the contrary, no distinct protein band was visible in the mutant’s supernatant sample t_e,S,Q204K_, and a focused, intense band was visible in the pellet sample (t_e,P,Q204K_).

### 3.4. NspER1-L1,5 Mutant Q204K: Crystallization Studies with Increasing Concentrations of Host Cell Protein (HCP)

Since technical protein crystallization is usually performed in the presence of HCP, µL-scale crystallization studies were performed with *Nsp*ER1-L1,5 mutant Q204K and increasing amounts of HCP (dialyzed IMAC flow-through, see Section 2.3). The tested HCP proportions and the varying PEG concentrations are listed in Table 3, along with the detected crystallization start and duration until crystallization equilibrium determined by evaluating photomicrographs.

**Figure 6:**
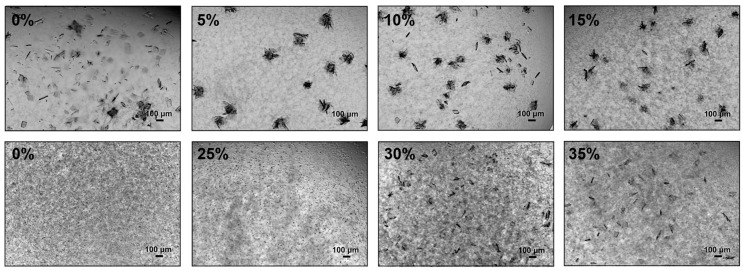
Photomicrographs of *Nsp*ER1-L1,5 mutant Q204K (5 g L^−1^) µL-batch crystallization with increasing amounts of HCP: 0–15% (*w*/*v*) and 150 g L^−1^ PEG 6000 (**top row**) and 0–35% (*w*/*v*) and 250 g L^−1^ PEG 6000 (**bottom row**) after 40 h.

Photomicrographs of *Nsp*ER1-L1,5 mutant Q204K batch crystallization with increasing amounts of HCP (0–35% (*w*/*v*)), which resulted in crystals within 40 h, are depicted below (Figure 6).

The addition of 5–15% (*w*/*v*) HCP to the crystallization mix with 150 g L^−1^ PEG 6000 resulted in heterogeneous crystallization with star-shaped crystals. For HCP proportions ≥20% (*w*/*v*), no crystal growth was documented with 150 g L^−1^ PEG 6000 within 40 h. Hence, the crystallization agent concentration was increased to 250 g L^−1^ PEG 6000 (Figure 6, bottom row). With 250 g L^−1^ PEG 6000, crystallization could be detected with up to 35% (*w*/*v*) HCP. The crystal size increased with increasing HCP concentration. WithoutHCP (0%), microcrystals were visible. The crystal size increased to 150 µm at 35%, with increased protein aggregation (0–35%) compared to 150 g L^−1^ PEG 6000. Crystallization with ≥40% (*w*/*v*) HCP did not result in crystals in static µL-batch crystallization studies.

**Figure 7:**
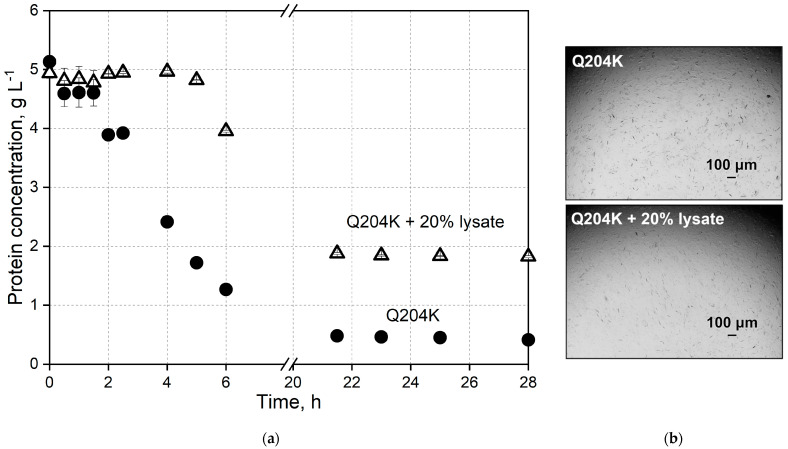
Stirred crystallization of the purified *Nsp*ER1-L1,5 mutant Q204K and Q204K with 20% (*w*/*v*) HCP. (**a**) Supernatant protein concentration of the *Nsp*ER1-L1,5 mutant Q204K with and without HCP during crystallization from an IMAC-purified protein solution in stirred crystallizers (c_0_ = 5 g L^−1^, V = 5 mL, n_Stirrer_ = 150 min^−1^, T = 20 °C, 250 g L^−1^ PEG 6000). (**b**) Photomicrographs of the *Nsp*ER1-L1,5 mutant Q204K with and without HCP taken after 21.5 h of crystallization (10-fold diluted with the protein buffer).

To scale up the *Nsp*ER1-L1,5 crystallization with HCP, purified *Nsp*ER1-L1,5 mutant Q204K was crystallized in parallel 5 mL stirred crystallizers, concurrent with an approach using 20% (*w*/*v*) HCP (Q204K + 20% *E. coli* lysate), as depicted below (Figure 7). The supernatant concentration for mutant Q204K started to decrease after 1.5 h to 1.3 g L^−1^ within the next 4.5 h, with an equilibrium concentration of 0.4 g L^−1^ reached within 21.5 h (92.2% yield). Contrary to the Q204K mutant, the addition of 20% (*w*/*v*) HCP decelerated the crystallization kinetics, leading to a reduction in supernatant concentration after 4 h to 4.0 g L^−1^ within the next 2 h and an equilibrium concentration of 1.8 g L^−1^ reached within 21.5 h (84.0% yield, adjusted for lysate).

### 3.5. X-Ray Structure Analysis of Selected NspER1-L1,5 Mutants

**Figure 8:**
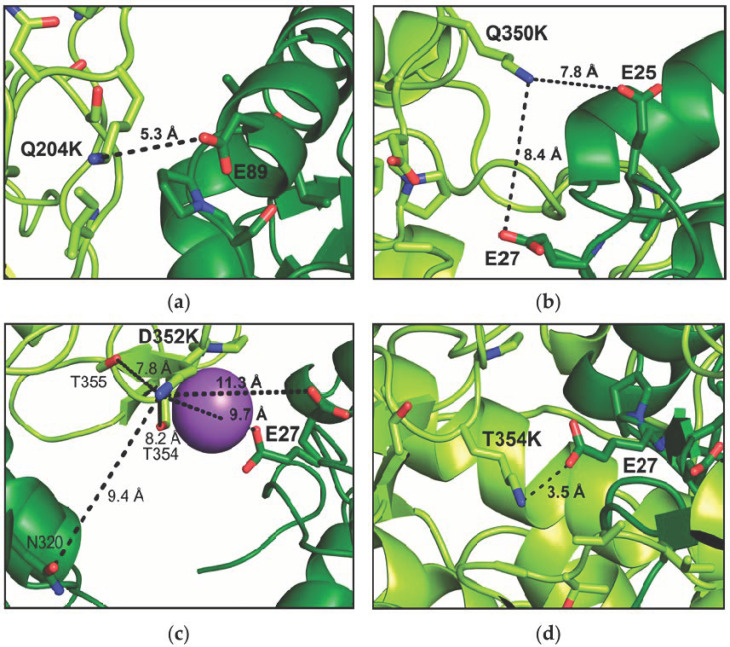
Illustration of the *Nsp*ER1-L1,5 crystal contact at positions (**a**) Q204K, (**b**) Q350K, (**c**) D352K, and (**d**) T354K (Neighboring monomers are differentiated in color by light/dark green). The distances to the intermolecular interaction partners E89 and E25/27 were calculated using PyMOL (v.2.3 [45]).

To validate the effect of the introduced single amino acid exchanges on a molecular level, the X-ray structure of the crystallized *Nsp*ER1-L1,5 mutants was analyzed. The root-mean-square deviation (RMSD) values after the alignment of each mutant with the *Nsp*ER1-L1,5 wild type are given in Supplementary Table S3, showing an excellent structural agreement between the variants. Additionally, an alignment of the crystalline environment of each mutant with the wild type was performed. Mutants D352K and T354K crystallizing in the same space group P12_1_1 as the wild type showed, again, an excellent agreement in crystal packing. Mutants Q204K and Q350K crystallized in space group P2_1_2_1_2_1_, with the RMSD of the crystalline environment in this crystal packing indicating structural differences. However, as demonstrated by Figure 8, the crystal contacts at the mutation sites were present as derived from the *Nsp*ER1-L1,5 wild-type structure. Since the alignment of the *Nsp*ER1-L1,5 mutants revealed similar crystal contacts to the WT, this allowed a comparison between the crystallizability of the *Nsp*ER1-L1,5 variants and the inference regarding the mutated site and the change in crystallizability of the respective *Nsp*ER1-L1,5 mutants. *Nsp*ER1-L1,5 mutants Q204K, Q350K, D352K, and T354K led to the highest crystallizability. High-resolution X-ray datasets were collected for the crystals, and the structures of the mutants were refined. To elucidate the molecular basis for improved crystallizability, the respective crystal contacts were compared to the WT and are depicted below in Figure 8. The electron density map of the crystal contact positions 204 and 350–354 for the *Nsp*ER1-L1,5 wild type and mutants Q204K, Q350K, D352K, and T354K are available in Supplementary Figure S2.

The crystal contact at positions Q204K (Figure 8a) and Q350K (Figure 8b) both reveal an approximation of the intermolecular partners E89 (5.3 Å) and E27 (7.8 Å), respectively, compared to the WT. The crystal contact at position D352K (Figure 8c) is not conform to expectations and is quite complex, with more than one amino acid residue interacting with D352K within approx. 10 Å. A sodium ion (lilac) also seemed to participate in the interaction complex. The distance of 3.5 Å between the carboxy group of E27 and the amino group of mutant T354K indicates the formation of a salt bridge (Figure 8d). Introducing a lysine at any of the neighboring *Nsp*ER1-L1,5 positions Q350, (D352), and T354 led to an interaction with the intermolecular amino acid residue of E25/27.

## 4. Discussion

In this study, the *Nostoc* sp. PCC 1720 ene reductase variant *Nsp*ER1-L1,5 was utilized as an example protein for the rational introduction of Lys–Glu interactions at crystal contacts to demonstrate the impact of rational single amino acid exchanges on the crystallizability of proteins and to enhance protein crystallization. For the nine pre-selected *Nsp*ER1-L1,5 mutants intended to show enhanced electrostatic interactions, the following outcome was observed during the protein processing. For 100% of the tested *Nsp*ER1-L1,5 mutants (9/9), the mutation, heterologous protein production, and protein processing (IMAC purification and dialysis) were successfully performed. The enzymatic activity of the nine tested *Nsp*ER1-L1,5 mutants was preserved (Supplementary Figure S1). This demonstrates a reliable and reproducible experimental setup for *Nsp*ER1-L1,5 according to the established crystal contact engineering experiments with *Lb*ADH/*Lk*ADH [19,20]. In the µL-batch experiments, 78% (7/9) of the preselected *Nsp*ER1-L1,5 mutants crystallized: Q171E, Q204K, Q263K, D280K, Q350K, D352K, and T354K. Out of these seven, three mutants (43%) crystallized significantly better than the *Nsp*ER1-L1,5 WT, as demonstrated by five different parameters defined for enhanced crystallizability (see Section 1): Q204K (5/5), D352K (2/5), and T354K (2/5). It is noteworthy that the nucleation window of Q204K (compared to the WT) not only extended to reduced protein and crystallization agent concentrations (Figure 2), but also to different buffers (Figure 3).

As described in the Introduction, the increased crystallizability due to the introduced Lys–Glu interactions has already been demonstrated for two non-ER-homologous enzymes, *Lb*ADH/*Lk*ADH, as shown in several studies [17,19,20]. These studies, combined with the findings from this research with *Nsp*ER1-L1,5, a non-ADH-homologous enzyme, support a general strategy to enhance crystallizability by introducing electrostatic Lys–Glu interactions. The approach is further confirmed by a large survey of crystal contacts, which concluded that the Lys–Glu interaction is one of the most favored pairwise contacts—both in oligomer and crystal contacts [47].

Validating the molecular structure of the *Nsp*ER1-L1,5 mutants, for 67% (4/6) of the analyzed crystal structures, the correct intermolecular interaction partner was assumed (except for D352K; and Q263K: E146 instead of E44, data not shown). Yet, the correct interaction type was predicted in all cases: electrostatic interaction (via Lys–Glu). This demonstrates the reliability of the rational introduction of electrostatic interactions as a crystal contact engineering strategy. Distances between the participating functional groups of salt bridges were defined to be ≤4 Å, which could be applied to T354K, explaining its enhanced crystallizability. For mutants Q204K (4.5 Å) and Q350K (7.8 Å), the formation of a long-range ion pair between Lys–Glu, which could exhibit an interaction within a range of 5–10 Å [48], could account for the enhanced crystallizability, especially for mutant Q204K. The neighboring *Nsp*ER1-L1,5 mutants Q350K, D352K, and T354K led to an interaction with intermolecular E25/27 (Figure 8) and an increase in crystallizability (Section 3.2, Section 3.3 and Section 3.4), with the strongest impact for T354K. This indicates that introducing electrostatic interactions is not limited to single positions but can also be applied to suitable neighboring amino acid residues, making the approach even more applicable. It should be pointed out that the introduction of a positive charge led to increased crystallizability in four out of the seven *Nsp*ER1-L1,5 mutants (Table 2), whereas the introduction of a negative charge had an adverse (1/2) or no effect (1/2). Further, it should be noted that the introduction of charged amino acids may not be ideal for all amino acid groups, as the exchange of alanine or valine with hydrophobic side chains for lysine (*Nsp*ER1-L1,5 mutants A264K, V344E; Table 2) did not result in crystals.

Although technical crystallization was introduced and highlighted as an emerging purification method, Schmidt et al. pointed out bottlenecks, such as crystallization from impure solutions and scaling-up from static to dynamic crystallization [5]. This study demonstrates that dynamic crystallization of purified *Nsp*ER1-L1,5 mutant Q204K can be performed reproducibly (Figure 4 and Figure 8). When the pure Q204K protein solution is spiked with 20% (*w*/*v*) HCP and crystallized in a stirred 5 mL crystallizer, the target protein equilibrium concentration approximately doubled from 0.4 g L^−1^ to 0.8 g L^−1^ (Figure 7, adjusted for the HCP). Nevertheless, the upscaling experiments resulted in reproducible batch crystallization for *Nsp*ER1-L1,5 mutant Q204K with a slight reduction in yield to 84% compared to the pure protein solution with a 92.2% yield. A similar study with an identical technical setup using lysozyme—a general reference protein for crystallization—reached protein solutions spiked with up to 15% (*w*/*v*) HCP for stirred mL-batch crystallization and up to 93.7% yield [49].

As the anything-but chromatography approach for bio(pharmaceutical) separation techniques has sparked interest recently (reviewed in [2,50]), advances in protein crystallization have rendered this method a potential alternative to conventional packed-bed chromatography. (Stirred) batch crystallization from *E. coli* cell lysate would render the time-consuming and cost-intensive [24,51,52] protein purification step via chromatography dispensable, which has been successfully demonstrated for *Lb*ADH/*Lk*ADH [19,20], homoserine oxygen-acetyltransferase [53], and on a 1-liter scale for a therapeutic monoclonal IgG1 antibody (88–90% yield, 98.5% purity) [54]. Thus, the increase inHCP proportion for batch crystallization with *Nsp*ER1-L1,5 mutant Q204K was investigated. Crystallization of *Nsp*ER1-L1,5 mutant Q204K was successfully performed with up to 35% (*w*/*v*) HCP in static µL-batch experiments, whereas the WT did not crystallize.

## 5. Conclusions

This study demonstrates that rational crystal contact engineering approaches can be transferred successfully between non-homologous enzymes (ADH to ER). This indicates that a more general approach to enhance protein crystallization by rational crystal contact engineering could be successfully developed. This impacts the technical applicability of industrial protein crystallization, raising even more interest as an alternative to conventional chromatography in downstream processing as a low-cost and time-saving purification step [51]. Crystalline proteins also offer further advantages for the biopharmaceutical industry, as crystalline therapeutics are characterized by high stability and extended shelf life, as well as a high drug concentration at low viscosity for slow and sustained release *via* subcutaneous administration [12].

## Figures and Tables

**Table 1:** Rational selection of *Nsp*ER1-L1,5 positions for mutations at crystal contacts within a 6 Å distance. The introduced amino acid should interact with the potential partner listed, resulting in an electrostatic Glu–Lys interaction. *Nsp*ER1-L1,5 mutants were generated, and the distances were calculated *in silico* using PyMOL (v.2.3 [45]).

Mutant	Potential Interaction Partner	Distance (*In Silico*), Å
Q171E	K139	4.6
Q204K	E89	5.2
Q263K	E44	8.5
A264K	E44	3.1
D280K	E340	5.3
V344E	K139	5.4
Q350K	E25	5.2
D352K	E27	5.3
T354K	E27	3.8

**Table 2:** Evaluation of the µL-batch crystallization experiments for the purified *Nsp*ER1-L1,5 wild type (WT) and mutants (Mut) Q171E, Q204K, Q263K, D280K, and Q350K. A264K and V344E did not crystallize under the tested conditions within 50 h (/). The *Nsp*ER1-L1,5 variants were evaluated regarding the crystallization start and the time until crystallization equilibrium was reached at the lowest common crystallization condition (*) and the lowest PEG or protein concentration tested (50 mM Tris/HCl, 0.2 M NH_4_Cl, 5 mM CaCl_2_, 100–300 g L^−1^ PEG 6000, pH 8.5) at which crystals were detected. The results of these three parameters are summarized in the rightmost column, indicating whether the mutant proteins crystallized better (+), similar (0), or inferior (−) to the WT.

Mutant	Crystallization Start t_0_, Mut/WT, h (at*)	Crystallization Equilibrium, h (at*)	Lowest Protein + PEG 6000 Concentration for Mut/WT, g L^−1^	+/0/− WT
Q171E	6.5/2.5	50/>50	5 + 150	–
Q204K	0.5/30	6.5/>50	3.75 + 150	++
Q263K	0/0.5	30/>30	10 + 150/5 + 150	0
A264K	/	/	/	/
D280K	35/2	>50/>50	5 + 150	−
V344E	/	/	/	/
Q350K	0.5/2.5	45/>50	5 + 150	+
D352K	2.5/5.5	45/>50	5 + 150	+
T354K	2/2	45/>50	5 + 150; 10 + 100/5 + 150	+

**Table 3:** Crystallization studies with *Nsp*ER1-L1,5 mutant Q204K (5 g L^−1^) and the increasing amounts of HCP (0–35% (*w*/*v*)), combined with 150 g L^−1^ or 250 g L^−1^ PEG 6000 in the crystallization buffer.

Lysate Proportion, %	PEG 6000 Concentration, g L^−1^	Crystallization Start t_0_, h	Crystallization Equilibrium, h
0	150	2.0	30.0
5	150	0.5	>40
10	150	0.5	>40
15	150	0.5	>40
20	150	/	/
0	250	1.0	1.5
25	250	1.5	18.0
30	250	2.5	35.5
35	250	4.0	>40
40	250	/	/

## Data Availability

All X-ray crystal structure data in this study were deposited and are available in the Protein Data Bank under the identification codes 9QGB (*Nsp*ER1-L1,5 wild type), 9QGC (mutant Q204K), 9QGD (mutant Q350K), 9QGE (mutant D352K), and 9QGF (mutant T354K). All the other data generated or analyzed during this study are included in this article and the Supplementary Material or are available from the corresponding author upon reasonable request.

## Supplementary Materials

The following supporting information can be downloaded at: https://www.mdpi.com/article/10.3390/biom15040467/s1, Figure S1: Maximum enzymatic activity of *Nsp*ER1-L1,5 mutants Q171E, Q204K, Q263K, A264K, D280K, V344E, D350K, D352K, and T354K relative to the *Nsp*ER1-L1,5 wild type; Figure S2: Electron density map (grey) of *Nsp*ER1-L1,5 crystal contact positions (a) Q204 and (b) 350–354, as well as of the respective mutants (c) Q204K, (d) Q350K, (e) D352K, and (f) T354K with well-defined electron density of the lysine and glutamic acid side chains for Q204K and T354K. Small density spheres represent water molecules. The map represents the structure factor amplitude difference 2F_o_–F_c_ with a contour level of 1.0 σ (calculated with REFMAC [44]); Table S1: Forward (5′►3′) and reverse (3′►5′) oligonucleotide sequences for the site-directed mutagenesis of the selected *Nsp*ER1-L1,5 mutants via QuikChange PCR; Table S2: Data collection and refinement statistics of X-ray diffraction experiments of crystals from the *Nsp*ER1-L1,5 wild type and mutants Q204K, Q350K, D352K, and T354K; Table S3: List of the root-mean-square deviation (RMSD) of Cα atoms (Å) and chain alignments for the *Nsp*ER1-L1,5 wild type and mutants Q204K, Q350K, D352K, and T354K.
