# Supplementary material for: Rational Introduction of Electrostatic Interactions at Crystal Contacts to Enhance Protein Crystallization of an Ene Reductase
Source: Biomolecules. 2025 Mar 22;15(4):467. doi: 10.3390/biom15040467 (PMC12024682; doi:10.3390/biom15040467)
Supplement: Supplementary file 1 [file biomolecules-15-00467-s001.zip › biomolecules-3492531-supplementary.pdf]

Supplementary Material for

Rational Introduction of Electrostatic Interactions at Crystal Contacts to Enhance Protein Crystallization of an Ene Reductase

Brigitte Walla [1], Anna Maslakova [1], Daniel Bischoff [1], Robert Janowski [2], Dierk Niessing [2,3] and Dirk Weuster-Botz [1,*]

[1] Biochemical Engineering, Department of Energy and Process Engineering, TUM School of Engineering and Design, Technical University of Munich, Boltzmannstraße 15, 85748 Garching, Germany
[2] Molecular Targets and Therapeutics Center, Institute of Structural Biology, Helmholtz Zentrum München, Ingolstädter Landstraße 1, 85764 Neuherberg, Germany
[3] Institute of Pharmaceutical Biotechnology, Ulm University, James-Franck-Ring N27, 89081 Ulm, Germany
* Correspondence: dirk.weuster-botz@tum.de

Table S1. Forward (5'►3') and reverse (3'►5') oligonucleotide sequences for the site-directed mutagenesis of the selected *Nsp*ER1-L1,5 mutants *via* QuikChange PCR. Oligonucleotides were designed according to Zheng et al. [1] (mutation in bold).

***Nsp*ER1-L1,5 mutant**	**5' ► 3'**	**3' ► 5'**
Q171E	caatatcgc**gaa**ggggctgcaaac	gcccc**ttc**gcgatattgttcgac
Q204K	gacgaat**aaa**cgtacagacg	gtacg**ttt**attcgtcccatc
Q263K	gtagct**aaa**gcactgaaccgcttc	cagtgc**ttt**agctacgtaacc
A264K	gctcaa**aaa**ctgaaccgcttc	gttcag**ttt**ttgagctacgtaacc
D280K	gaagctata**aaa**gcagacattagac	gtctgc**ttt**tatagcttcaaag
V344E	ctagag**gaa**aacgcaccactc	gtgcgtt**ttc**ctctaggcgttc
D350K	cactcaat**aaa**gcagaccccacaac	gtctgc**ttt**attgagtggtgcgttc
D352K	caatcaggca**aaa**cccacaacct	gtggg**ttt**tgcctgattgagtggtg
T354K	gacccca**a**aaccttttatggtgg	aaaggtt**t**tggggtctgcctgattg

Table S2. Data collection and refinement statistics of X-ray diffraction experiments of crystals from the *Nsp*ER1-L1,5 wild type and mutants Q204K, Q350K, D352K, T354K (values in parentheses are for highest resolution shell).

**Variant**	**Wild type**	**Q204K**	**Q350K**	**D352K**	**T354K**
**Data collection**					
**PDB ID**	9QGB	9QGC	9QGD	9QGE	9QGF
**Beamline**	SLS Beamline X06SA	PETRA III, DESY BEAMLINE P11	PETRA III, DESY BEAMLINE P11	PETRA III, DESY BEAMLINE P11	PETRA III, DESY BEAMLINE P11
**Wavelength, Å**	1.00	1.03	1.03	1.03	1.03
**Space group**	P 1 21 1	P 21 21 21	P 21 21 21	P 1 21 1	P 1 21 1
**Cell constants a, b, c, α, β, γ**	55.2Å 67.8Å 91.5Å 90.0° 93.5° 90.0°	55.4Å 68.6Å 90.0Å 90.0° 90.0° 90.0°	55.3Å 68.4Å 89.9Å 90.0° 90.0° 90.0°	55.4Å 68.2Å 91.63Å 90.0° 94.1° 90.0°	55.5Å 68.1Å 91.71Å 90.0° 93.7° 90.0°
**Chains per asymmetric unit**	2	1	1	2	2
**Resolution, Å**	48.8-1.27 (1.34-1.27)	48.8-1.24 (1.31-1.24)	48.8-1.24 (1.31-1.24)	48.8-1.43 (1.52-1.43)	48.8-1.44 (1.53-1.44)
**CC (1/2)**	1.00 (0.65)	1.00 (0.52)	1.00 (0.73)	1.00 (0.57)	1.00 (0.53)
**Variant**	**Wild type**	**Q204K**	**Q350K**	**D352K**	**T354K**
**Completeness, %**	97.5 (90.9)	99.5 (97.0)	98.5 (91.3)	98.3 (96.8)	98.8 (98.3)

**Redundancy**	6.75 (5.60)	9.31 (6.66)	12.57 (9.88)	5.12 (4.98)	6.84 (6.54)
**Refinement**					
**Resolution, Å**	1.27	1.24	1.24	1.43	1.44
**R_{work} / R_{free}**	0.13 / 0.16	0.13 / 0.16	0.12 / 0.15	0.15 / 0.20	0.14 / 0.19
**No. atoms**	7065	3482	3575	6661	6781
**Protein**	5743	2947	2987	5706	5749
**Water**	1160	472	532	882	900
**Other**	162	63	56	73	132
**B-factors**					
**Overall**	16.0	18.0	20.0	17.0	17.0
**Protein main chain**	11.96	14.33	15.28	14.14	13.94
**Protein side chain**	14.09	16.61	17.73	16.02	15.82
**R.m.s. deviations**					
**Bond lengths, Å**	0.01	0.01	0.01	0.01	0.01
**Bond angles, °**	1.91	1.92	2.10	1.80	1.83
**Ramachandran plot**					
**Most favored, %**	92.72	93.00	93.56	92.86	92.84
**Additional allowed, %**	6.72	5.88	5.60	6.16	6.60

Table S3. List of the root-mean-square deviation (RMSD) of Cα atoms (Å) and chain alignments for the *Nsp*ER1-L1,5 wild type and mutants Q204K, Q350K, D352K, and T354K.

RMSD (Å)	Wild type (A,B)	Q204K (D)	Q350K (D)	D352K (A,B)	T354K (A,B)
**Crystal environment alignment**					
**wild type (A)**	0, 2.84	9.17	9.15	2.92, 0.34	2.84, 0.27
**wild type (B)**	2.84, 0	10.2	10.19	0.33, 2.92	0.27, 2.84
**Monomer alignment**					
**wild type (A)**	0.00, 0.11	0.10	0.11	0.11, 0.07	0.11, 0.05

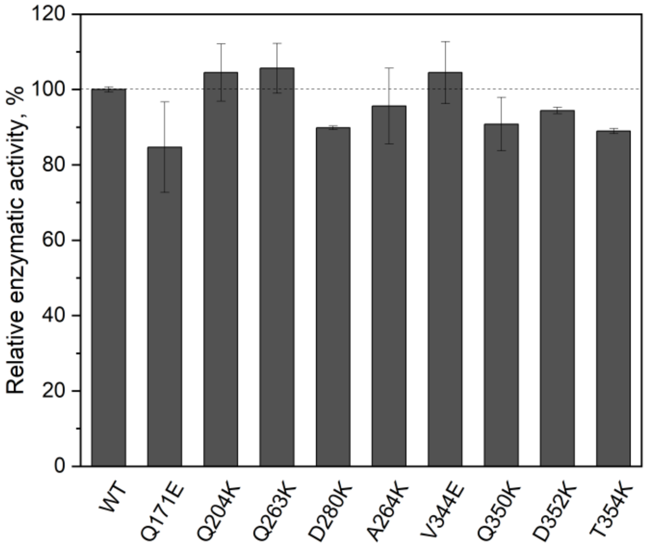

Figure S1. Maximum enzymatic activity of *Nsp*ER1-L1,5 mutants Q171E, Q204K, Q263K, A264K, D280K, V344E, D350K, D352K, and T354K relative to the *Nsp*ER1-L1,5 wild type (WT, set to 100%). IMAC-purified protein solutions of *Nsp*ER1-L1,5 variants were adjusted to 60 mg L^{-1}. Enzymatic activity was measured spectrophotometrically for 10 min at 340 nm (30 °C, every 6 sec) with addition of 180 µL buffer (120 mM NaH_2PO_4/ Na_2HPO_4, pH 7.0) containing 0.2 mM NADH and 10 mM maleimide to a final volume of 200 µL (n = 5).

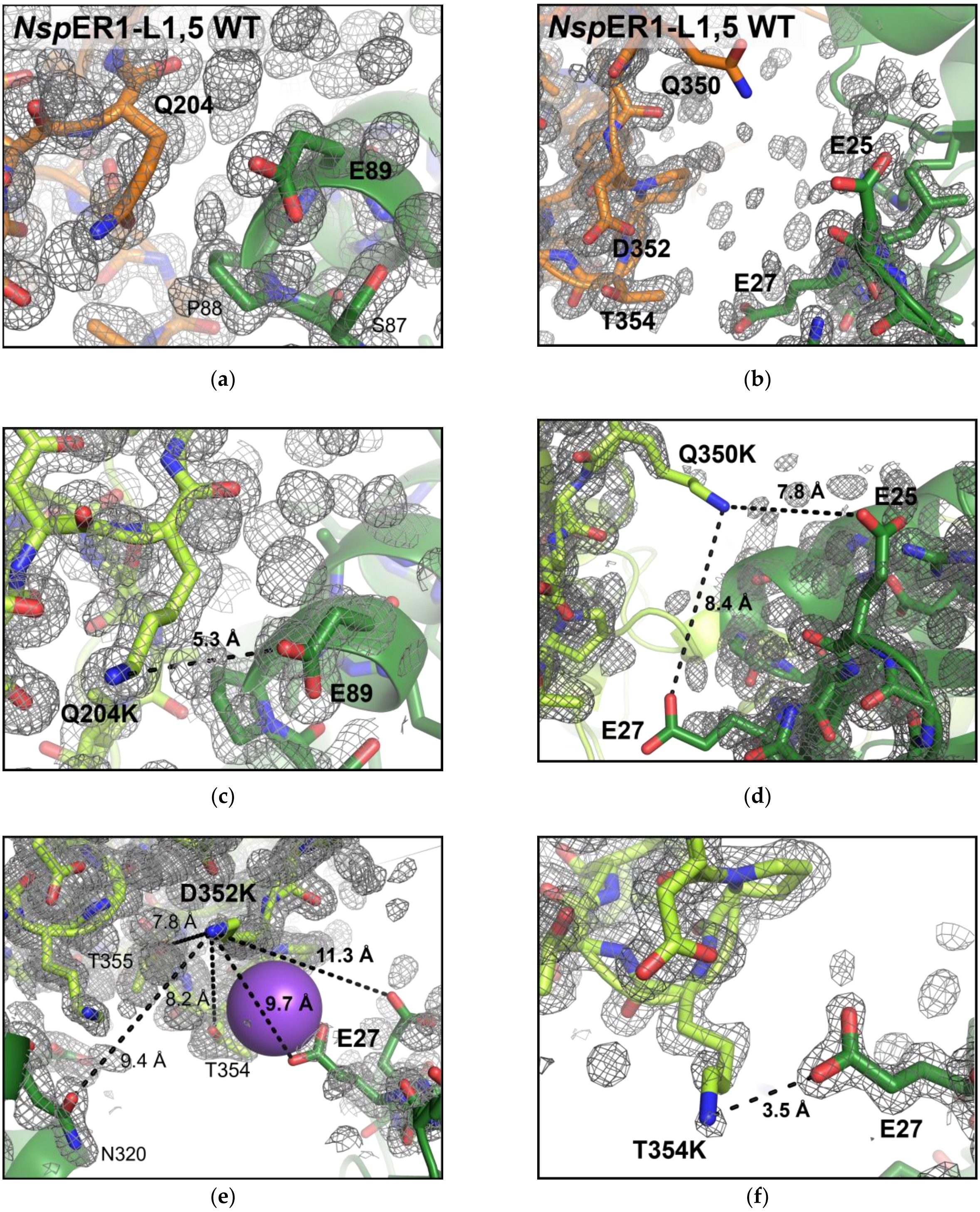

Figure S2. Electron density map (grey) of *Nsp*ER1-L1,5 crystal contact positions (**a**) Q204 and (**b**) 350-354, as well as of the respective mutants (**c**) Q204K, (**d**) Q350K, (**e**) D352K, and (**f**) T354K with well-defined electron density of the lysine and glutamic acid side chain for Q204K and T354K. Small density spheres represent water molecules. The map represents the structure factor amplitude difference $2F_o - F_c$ with a contour level of 1.0 σ (calculated with REFMAC [2]).

Reference

1. Zheng, L., Baumann, U., and Reymond, J. L. "An efficient one-step site-directed and site-saturation mutagenesis protocol." *Nucleic Acids Res* 32, no. 14 (2004): e115. 10.1093/nar/gnh110
2. Murshudov, G. N., Vagin, A. A., and Dodson, E. J. "Refinement of macromolecular structures by the maximum-likelihood method." *Acta Crystallographica Section D* 53, no. 3 (1997): 240-55. doi:10.1107/S0907444996012255
