# Supplementary figures and images for: Rational Introduction of Electrostatic Interactions at Crystal Contacts to Enhance Protein Crystallization of an Ene Reductase
Source: Biomolecules. 2025 Mar 22;15(4):467. doi: 10.3390/biom15040467 (PMC12024682; doi:10.3390/biom15040467)

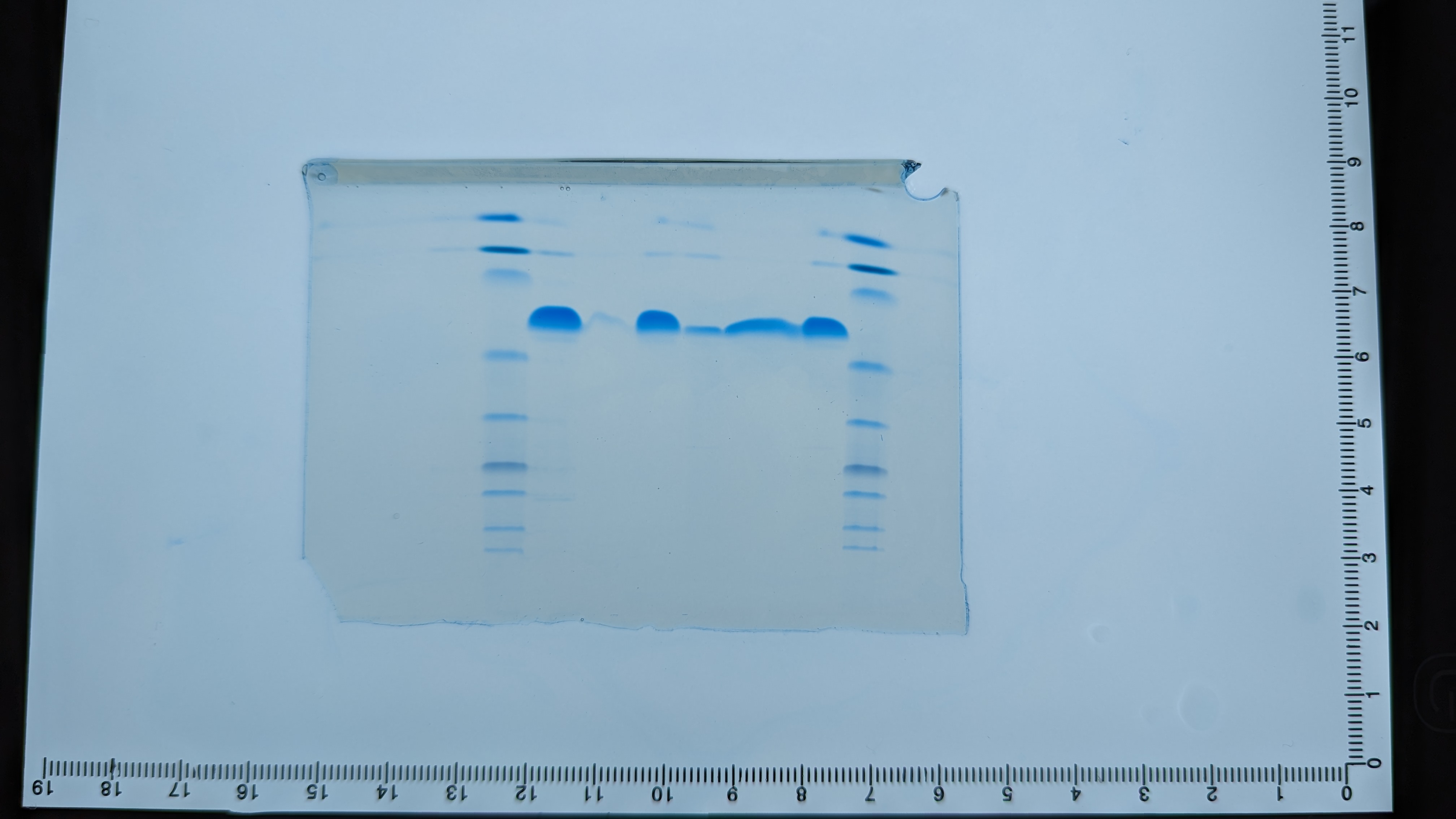

Supplement: Supplementary file 1 [file biomolecules-15-00467-s001.zip › biomolecules-3492531-original-images.jpg]
